# Tracking the Global Prevalence and Distribution of the AmpC-β-Lactamase-Carrying pESM Megaplasmid in *Salmonella* Minnesota

**DOI:** 10.3390/genes17101230

**Published:** 2026-10-03

**Authors:** Anamaria M. P. Dos Santos, Ana Beatriz Portes, Hannay Crystynah Almeida De Souza, Pedro Panzenhagen, Arlen Carvalho de Oliveira Almeida, Rafaela G. Ferrari, Carlos A. Conte-Junior

**Affiliations:** 1 Center for Food Analysis (NAL), Technological Development Support Laboratory (LADETEC), Federal University of Rio de Janeiro (UFRJ), Cidade Universitária, Rio de Janeiro 21941-598, RJ, Brazilconte@iq.ufrj.br (C.A.C.-J.); 2 Laboratory of Advanced Analysis in Biochemistry and Molecular Biology (LAABBM), Department of Biochemistry, Federal University of Rio de Janeiro (UFRJ), Cidade Universitária, Rio de Janeiro 21941-909, RJ, Brazil; 3 Graduate Program in Veterinary Hygiene (PGHIGVET), Faculty of Veterinary Medicine, Fluminense Federal University (UFF), Vital Brazil Filho, Niterói 24230-340, RJ, Brazil; 4 Laboratory of Microorganism Structure, Department of General Microbiology, Institute of Microbiology Paulo De Góes (IMPG), Federal University of Rio de Janeiro, Rio de Janeiro 21941-902, RJ, Brazil; 5 Graduate Program in Biochemistry (PPGBq), Institute of Chemistry (IQ), Federal University of Rio de Janeiro (UFRJ), Cidade Universitária, Rio de Janeiro 21941-909, RJ, Brazil; 6 Graduate Program in Food Science (PPGCAL), Institute of Chemistry (IQ), Federal University of Rio de Janeiro (UFRJ), Cidade Universitária, Rio de Janeiro 21941-909, RJ, Brazil; 7 Analytical and Molecular Laboratory Center (CLAn), Institute of Chemistry (IQ), Federal University of Rio de Janeiro (UFRJ), Cidade Universitária, Rio de Janeiro 21941-909, RJ, Brazil

**Keywords:** plasmid, *Salmonella* spp., Beta-lactam, β-lactam, antimicrobial resistance, antibiotic resistance, mobile genetic element

## Abstract

**Background/Objectives**: AmpC enzymes, encoded by the *bla*_CMY-2_ gene, confer resistance to a broad range of β-lactam antibiotics, including first-, second-, and third-generation cephalosporins, as well as β-lactam/β-lactamase inhibitor combinations. These genes can be located either on the chromosome or plasmids, with the plasmid-borne form posing a greater risk due to its potential for rapid dissemination via horizontal gene transfer. The pESM megaplasmid, identified in *Salmonella* Minnesota isolates, was characterized as carrying the *bla*_CMY-2_ gene, underscoring the urgent need for surveillance and control measures to address the threat of widespread resistance. **Methods**: In this *in silico* study, we analyzed the frequency and geographic distribution of pESM among publicly available *S.* Minnesota genomes over the last 20 years. **Results**: A total of 935 sequences were identified, of which 648 (69.3%) carried pESM. The countries with the highest number of positive isolates were Saudi Arabia (*n* = 303) and Brazil (*n* = 155), with a predominance of samples derived from poultry (*n* = 423; 65.27%). Genes involved in yersiniabactin synthesis and transport (*ybtT*, *ybtU*, *ybtS*, and *ybtX*) exhibited high proportion within the pESM-positive genomes, as did the *merA* gene, which is associated with mercury tolerance. Multi-locus sequence type (MLST) analysis showed that only ST548 and ST9594 carried the pESM. Forty distinct plasmid incompatibility groups were identified, with the most frequent being IncA/C2 (79.79%), followed by Col440I (74.97%), IncR (9.95%), IncI-1 (6.74%), ColpVC (8.02%), ColRNAI (5.45%), IncQ1 (4.71%), and IncFII(S) (4.28%. All pESM-positive genomes carried *bla*_CMY-2_, with high co-occurrence of *sul2*, *tet(A)*, and the *mer* operon, indicating that putative pESM could mediate resistance to multiple antibiotics and heavy metals. Since 2015, the co-occurrence of these genes with pESM has increased, likely reflecting selection pressure and clonal expansion of successful lineages. Phylogenetic analysis revealed a recent clade primarily composed of poultry-derived isolates from Brazil and Saudi Arabia. Putative pESM is strongly associated with the IncA/C2 incompatibility group and the *qnrB19* gene, reinforcing its role in disseminating antimicrobial resistance. **Conclusions**: These findings suggest putative pESM is central in spreading multidrug resistance and virulence traits in *S.* Minnesota, especially in poultry environments. The plasmid’s prevalence in distinct sequence types and countries underscores its potential for horizontal transfer and global dissemination. Our results emphasize the need for enhanced surveillance of megaplasmids in foodborne pathogens and strategies to mitigate their spread.

## 1. Introduction

*Salmonella enterica* is one of the most prominent foodborne pathogens worldwide, being linked to high morbidity rates and responsible for diseases such as gastroenteritis, bacteremia, and enteric fever [1]. The transmission route typically occurs through the ingestion of contaminated products, and certain foods such as fresh produce, seafood, and nuts trigger widespread cross-border outbreaks [1]. Over 2,600 serovars of *S. enterica* have already been identified [2]; however, only a small part of them are effectively studied and associated with global human infections. Nevertheless, some serovars stand out and have emerged to impose localized and international threats, such as *S.* Heidelberg, *S.* Infantis, and *S.* Dublin [3,4,5].

*S.* Minnesota has emerged in the last few years as one of the most frequently isolated *S.* serovars in Brazilian poultry production systems [6,7,8,9]. Epidemiological studies have revealed a clonal lineage presenting multidrug resistance to cephalosporins, tetracycline, and sulphonamide, besides the high mercury tolerance due to the *mer* operon [6]. In a previous study, we revealed that most of these resistance genes were being disseminated through a unique megaplasmid of resistance and virulence, which was thought to be responsible for the widespread multidrug resistance of *S.* Minnesota in the Brazilian poultry chain [8]. Also, by phylogenetics and Bayesian analysis, we concluded that a sample from abroad likely underwent clonal expansion, promoting the *S.* Minnesota emergence in Brazil [8]. The plasmid for emergent *S.* Minnesota (pESM) (~210 kb) encodes multiple resistance genes—*sul2* (predicted sulphonamide resistance)*, tet*(A) (predicted tetracycline resistance), and *bla*_CMY-2_ (predicted cephalosporins resistance)—besides the *mer* operon and the yersiniabactin siderophore, related to mercury tolerance and iron uptake, respectively [8].

The structure of pESM is very similar to the plasmid for emergent *S.* Infantis (pESI), which has emerged in several countries in Europe and the United States, characterized by carrying at least three antibiotic resistance genes and several genes related to persistence in the environment [10]. Like pESI, pESM spreads through clonal expansion, potentially being horizontally transferred due to the presence of transfer (*tra*) genes. In our previous study, we demonstrated the presence of pESM in other countries besides Brazil [8]. However, the global dissemination of pESM and its role in the spread of AmpC β-lactamases were not explored in depth, as this was beyond the primary scope of that investigation.

Considering the possible risk associated with the global dissemination of this virulent and resistant megaplasmid, we investigated the prevalence and worldwide distribution of the pESM carrying the *bla*_CMY-2_ gene, which encodes AmpC β-lactamases and may confer resistance against first-, second-, and third-generation cephalosporins. We also evaluated the frequency of other “backbone or signature” genes related to pESM over the last 20 years and in different isolation sources and locations.

## 2. Methods

### 2.1. Data Collection and Putative pESM Megaplasmid Screening

We used the filtering tools available on the pathogen detection database of the National Center for Biotechnology Information (NCBI) to select the *S.* Minnesota genomes available on 28 October 2024. The “Run selector” SRA tool was applied to filter the Illumina runs and download the raw Illumina paired-end reads using the SRAToolkit v 2.11.3 using the -prefetch option. *De novo* assembly was performed using the SPAdes v 3.11 software, available within the Shovill v1.1.0 pipeline [11]. After assembly, we used SISTR (*Salmonella* in silico typing resource) v1.1.1 to confirm and select the *S.* Minnesota serovar [12]. To infer the presence of the plasmid for emergent *S.* Minnesota (pESM), the complete plasmid previously described by dos Santos et al. [8] (Accession number CP168004.1) was annotated using Prokka v1.14.6 software [13] and the gene’s FASTA annotation file was used to create a custom database in ABRicate v1.0.1, following the software instructions available in (https://github.com/tseemann/abricate, accessed on 28 October 2024). The custom database with the pESM gene information was locally aligned against the *S.* Minnesota genomes, with a minimum threshold of 95% identity and 60% coverage. Since the genomes evaluated consist of short-read sequences, identifying a megaplasmid is challenging due to technical issues; however, the extensive presence of most of the genes constituting the megaplasmid increases the likelihood that the genomes contain pESM. However, to increase this probability, 10 target genes were selected and locally aligned against the *S.* Minnesota genomes, based on the identification model proposed by Franco et al. [14] in a pESI study and the pESM study by dos Santos et al. [8] (Table 1). The genomes that presented nine of the 10 target genes and over 90% of the pESM gene composition were considered to have a putative pESM megaplasmid.

### 2.2. In Silico Characterization and Statistical Analysis

We used ABRicate v1.0.1 software (https://github.com/tseemann/abricate, accessed on 28 October 2024) to identify the virulence genes and plasmid replicons (minimum 95% identity and 60% coverage) by performing a local alignment against the VFDB (Virulence finder database) [15] and Plasmidfinder [16] databases, respectively. Antimicrobial genes were identified using the AMRFinderPlus v3.12.8 software [17]. We used the available NCBI metadata to access the year and source of genome isolation, and to evaluate the frequency of the AmpC-β-lactamase resistance gene *bla*_CMY-2_ in pESM-positive *S.* Minnesota genomes over the years. Furthermore, we analyzed the distribution of the pESM target genes and other relevant resistance and virulence genes to investigate their association with the presence of putative pESM over the years.

We explored the associations between resistance, virulence and replicon genes identified along *S.* Minnesota genomes and pESM, in relation to biological co-occurrence and the possible central involvement of pESM in the spread of resistance and virulence. Because pESM status was inferred from the presence of the target genes (Table 1) and of >90% of the pESM reference gene content (CP168004.1), genes belonging to either set were not subjected to hypothesis testing, since their association with pESM is expected by definition. For these genes, frequencies are reported descriptively as n/N (%) among pESM-positive and pESM-negative genomes. Inferential testing was restricted to genes absent from the pESM reference (*n* = 12) (Supplementary Table S3). For each of these genes, a Pearson’s chi-square (χ^2^) test of independence with Yates’ continuity correction was performed against pESM status. Fisher’s exact test was used when any expected count was <5. Only genomes with complete binary data from both variables were included. *p*-values were adjusted for multiple comparisons using the Benjamini–Hochberg false discovery rate (FDR) procedure [18], and associations were considered significant if they had an adjusted *p*-value < 0.05. The direction of each association was assessed using adjusted standardized residuals [19], calculated using the formula

$$Z=\frac{\left(O-E\right)}{\surd [E(1-\frac{R}{N})(1-\frac{C}{N})}$$

where “*O*” and “*E*” are the observed and expected frequencies, “*R*” and “*C*” are the corresponding row and column totals, and “*N*” is the number of isolates. Positive residuals indicate genes over-represented among pESM-positive isolates, and negative residuals indicate under-representation (Supplementary Table S3).

All statistical analyses were conducted in R v 4.3.1 using Rstudio v 4.3.1, ggplot2 v 4.0.3, and writexl v 2.0.1 packages.

### 2.3. Phylogenetics Analysis

The detection and prediction of whole-genome variants were executed using rapid haploid variant calling and core genome alignment—Snippy v4.6.0 (https://github.com/tseemann/snippy, accessed on 3 November 2024). The ATCC 49284 (accession CP019184.1) strain was employed to identify and eliminate recombinant regions within the core genome alignment to enhance the accuracy of phylogenetic reconstructions with Gubbins v3.2.1 [20]. Additionally, Phastaf v0.1.0 (https://github.com/tseemann/phastaf, accessed on 3 November 2024) and Barrnap v0.9 (https://github.com/tseemann/barrnap, accessed on 3 November 2024) were utilized for the identification and masking of phage regions and ribosomal RNA, respectively, in the reference genome.

The final high-quality core genome SNPs were extracted from the alignment file using SNP-sites v2.5.1 [21]. Subsequently, Iqtree v2.0.3 [22] was utilized to generate a maximum-likelihood phylogeny from this core genome alignment, employing a GTR + F+I + G4 substitution model with standard 1000 bootstrap replications to support the tree nodes [23]. The resulting tree was visualized using iTOL (Interactive Tree of Life) [24].

## 3. Results and Discussion

### 3.1. Distribution of Putative pESM Worldwide

**Figure 1:**
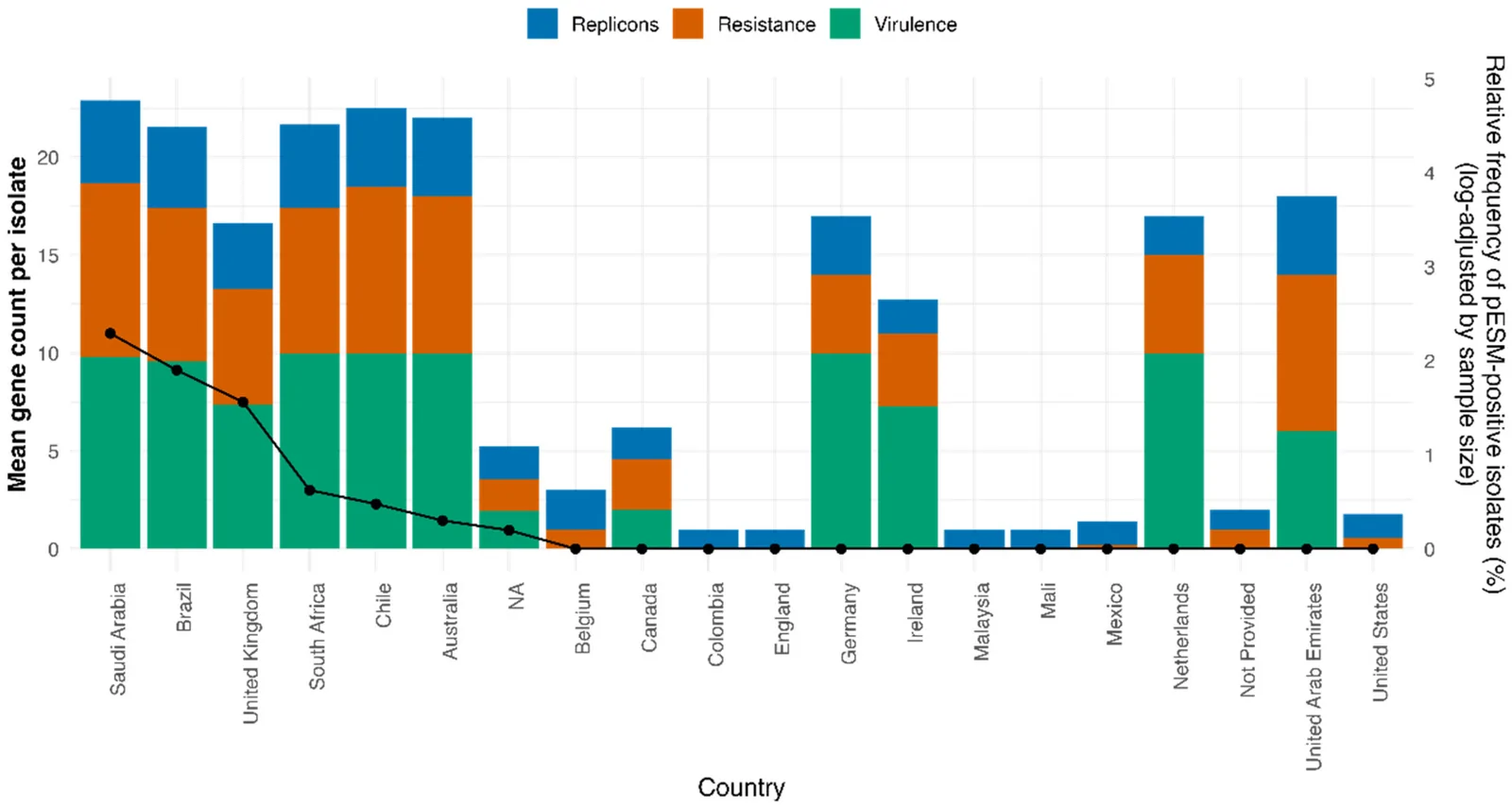
Average number of resistance, virulence, and plasmid replicon genes per isolate from each country, with the log-adjusted frequency of pESM-positive isolates (%) represented as black dots. The black dots represent the log-adjusted relative frequency of pESM-positive *Salmonella* Minnesota isolates correcting for sampling bias. Countries are ordered according to the adjusted frequency in descending order. To reduce distortions caused by an uneven number of genomes across countries, the proportion of pESM-positive isolates in each country was weighted by the base -10 logarithm of its sample size, as follows: adjusted frequency − (number of pESM-positive isolates/total number of isolates × log_10_(n + 1). Here, n is the total number of genomes from that country. This prevents countries with very few genomes (e.g., *n* = 1 or 2) from reaching values comparable to those countries with hundreds of genomes. NA is “Not available”.

The 935 selected genomes were isolated between 2000 and 2024 from various isolation sources, including animals (54.87%), food (23.32%), humans (11.76%), and the environment (0.8%) (Supplementary Material Table S1). The general dataset encompassed genomes from 18 countries (Supplementary Table S1, Figure 1), the most frequent being Saudi Arabia (*n* = 333/935), the United Kingdom (*n* = 276/935), and Brazil (*n* = 184/935).

When working with genomes deposited in public databases, it is essential to consider the sampling bias inherent to these repositories, as analysis is restricted to the available deposited data. Given the emergence of *S.* Minnesota specifically in the countries with the highest number of genomes (the United Kingdom [25], Saudi Arabia [26], and Brazil [8]), it was also expected that a larger proportion of genomes would be associated with these countries for epidemiological studies on this serovar.

Among the genomes evaluated, a large proportion, 648 (69.3%), presented the putative pESM. Those pESM-positive genomes were isolated from eight countries: Saudi Arabia (*n* = 303/648), Brazil (*n* = 155/648), the United Kingdom (*n* = 176/648), South Africa (*n* = 6/648), Chile (*n* = 2/648), Australia (*n* = 1/648), and four “Not provided” (Figure 1). Among these, 423 (65.27%) were isolated from poultry, exclusively from Brazil and Saudi Arabia (Supplementary Table S1).

The United Kingdom and Saudi Arabia have commercial relations with Brazil [27,28], and are among Brazil’s main importers of chicken meat. The presence of a putative pESM mainly in poultry-derived genomes from these countries may suggest that the plasmid could be disseminated internationally through chicken exports and undergo clonal expansion [29]. Our phylogenetic analyses demonstrate that the analyzed genomes share a common origin since they belong to the same monophyletic ancestor group.

Although our analysis focused on detecting putative pESM in *S.* Minnesota, it is likely that, similarly to pESI, this megaplasmid is not limited to a single serovar. Previous studies have shown that megaplasmids like pESI can be horizontally transferred to other *Salmonella* serovars under selective pressure [30,31]. For instance, pESI has been identified in *S.* Schwarzengrund in Japan [32] and *S.* Muenchen in Israel [33]. However, in Brazil, *S.* Minnesota is currently one of the most frequently isolated serovars in chicken [6,34], justifying the presence of a new megaplasmid adapted to this serovar in the Brazilian poultry production.

Something curious was the absence of putative pESM in genomes isolated from the United States, despite this country having the most extensive collection of genomes in the NCBI database [35]. Two main factors may also have contributed to this absence, beyond the database representation: the first is that *S.* Minnesota is not a dominant and emergent serovar in the USA [36]; the second is the dominance of other important serovars in the country, such as *S.* Infantis itself, which became dominant because of pESI carriage [37,38]. However, considering the selective pressures common to intensive poultry production systems, similarly to those observed in Brazil, it is plausible that the United States may also be subject to conditions favoring the future emergence of *S.* Minnesota carrying pESM.

### 3.2. Mercury Tolerance, Antimicrobial Resistance, and Virulence Profile

The high transmissibility of pESM, along with its carriage of multiple resistance genes, accelerates the emergence of multidrug-resistant bacteria. Given that pESM has a mosaic structure characterized by inserting components with different functions [8], it may acquire new genes in response to selective pressure. We observed that pESM is remarkable in poultry, which may tell us that poultry production is a central point for the clonal expansion of pESM among *S.* Minnesota [8]. A recent study demonstrated that the physicochemical characteristics of broiler litter, such as moisture, can directly influence the presence of *Salmonella* with large plasmids containing siderophores, like pESM and pESI [39]. As in the United States, Brazil tends to reuse broiler litter, which may reinforce selective pressure within these pathogens [40,41]. This suggests that the core issue lies in pre-harvest environments and not necessarily in slaughter and processing. Therefore, chicken-producing countries are more likely to export contaminated chickens. Brazil, being one of the largest producers and exporters of chicken meat globally [28], makes this possible dissemination a significant problem for global public health. If the characteristics of the litter are indeed responsible for the persistence of *Salmonella* carrying pESM and other megaplasmids, control measures such as reducing moisture or not reusing the litter should be taken. However, further studies relating environmental particularities and the high prevalence of multidrug-resistant *S.* Minnesota should be designed to understand the main selective pressures leading to the clonal expansion of these bacteria in Brazilian poultry production.

Genes involved in yersiniabactin synthesis and transport (*ybtT*, *ybtU*, *ybtS*, and *ybtX*) displayed a high proportion within the general dataset and among the pESM-positive genomes (Supplementary Table S3), reinforcing that yersiniabactin siderophore can be considered an important marker for identifying megaplasmids such as the pESM and the pESI in poultry environment [6,42,43]. This finding is particularly relevant in nutrient-limited environments such as the poultry gut and post-harvest food matrices, where the *ybt* operon is currently required [44,45]. The *merA* gene, related to mercury tolerance, was also proportionally high in pESM-positive genomes, corroborating the previous studies demonstrating that the high concentration of trace metals in the litter is also involved in selecting *Salmonella* with tolerance to mercury in poultry environments [39,46,47,48].

All the pESM-positive genomes (648/648, 100%) presented the *bla*_CMY-2_ gene related to the first, second, and third generations of cephalosporin resistance. We evaluated the co-occurrence of the *bla*_CMY-2_ gene with *sul2* (99.7%), *tet*(A) (99.7%), and *mer* operon (100%) in the pESM-positive genomes, related to sulphonamide, tetracycline, and mercury resistance, respectively. This co-occurrence indicates a likely mutual carriage of these genes by pESM-positive genomes, which can only be noted in genomes containing the megaplasmid. The high proportion of *bla*_CMY-2_ in pESM-positive genomes, as well as *sul2* and *tet*(A), could imply that pESM-positive genomes play a central role in maintaining cephalosporin, sulphonamide, and tetracycline resistance within clonal lineages of *S.* Minnesota. Therefore, *Salmonella* containing the pESM may restrict treatment due to the limited availability of antibiotic options.

**Figure 2:**
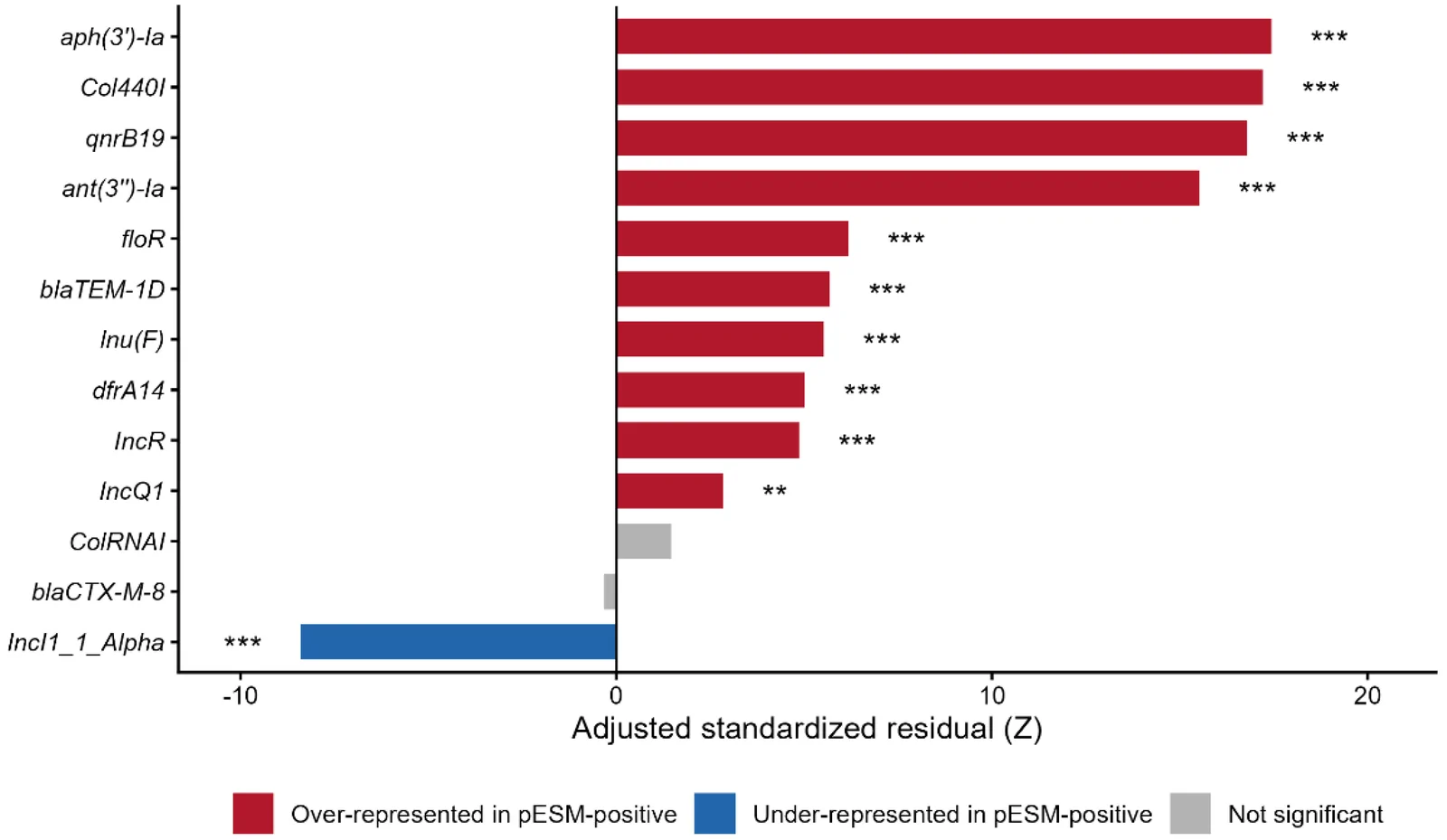
Gene/megaplasmid associations in *Salmonella* Minnesota isolates. Adjusted standardized residuals (Z) for genes absent from the pESM reference (CP168004.1), tested for association with pESM status. Positive values indicate over-representation among pESM-positive genomes and negative values indicate under-representation. Bar colors indicate the direction of significant associations. Gray bars are not significant. Asterisks denote Benjamini–Hochberg-adjusted *p*-values: ** *p* < 0.01, and *** *p* < 0.001.

For instance, other resistance genes, as well as replicons that were not present in the reference pESM gene content, were subjected to hypothesis testing to evaluate their association with pESM-positive genomes (Figure 2). Among the most significant associations are the aminoglycoside resistance genes *aph(3′)-Ia* and *ant(3″)-Ia*, the plasmid-mediated quinolone resistance gene (*qnrB19*), and the Col440I replicon (Figure 2), which, in addition to showing a highly significant association with pESM-positive genomes, were also present in a higher proportion of these genomes (Supplementary Table S3), which could indicate high co-occurrence. Other important resistance genes, such as *bla*_CTX-M-8_, *bla*_TEM-1D_ (resistance to β-lactams), *floR* (resistance to florfenicol), and *drfA14* (resistance to trimethoprim), also showed a significant association, despite their lower proportion (Supplementary Table S3). Since all genome assemblies in this study were derived from Illumina short reads, the identification of a ~210 kb megaplasmid can be challenging, given the intrinsic fragmentation of this type of sequencing [49]. By generating continuous reads spanning hundreds of kilobases, long-read sequencing represents an attractive alternative for investigating exceptionally large plasmids in genomics, which is particularly relevant given the emergence of megaplasmids in recent years [49]. Therefore, this type of analysis would also facilitate a better understanding of the evolution of these mobile genetic elements as vectors of antimicrobial resistance and virulence among pathogens, by facilitating the identification of these markers within a single plasmidial contig.

Recent studies in Brazil have demonstrated a high resistance rate to AmpC β-lactamases, mainly associated with exported and inland poultry meat [9,50,51,52]. In parallel, Campos et al. [9] revealed a high proportion of *bla*_CMY-2_ carried by IncA/C2 plasmids in *Salmonella* Minnesota and Heidelberg. This indicates that these replicons are related to disseminating β-lactam resistance among *Salmonella* isolated from poultry. Although the use of β-lactams as growth promoters in Brazil has been prohibited since 2009, this drug can still be used prophylactically in veterinary medicine [53], which might have influenced the increase in resistance to this class of antibiotics. Further studies are needed to better understand the distribution of AmpC β-lactamases and replicons.

**Figure 3:**
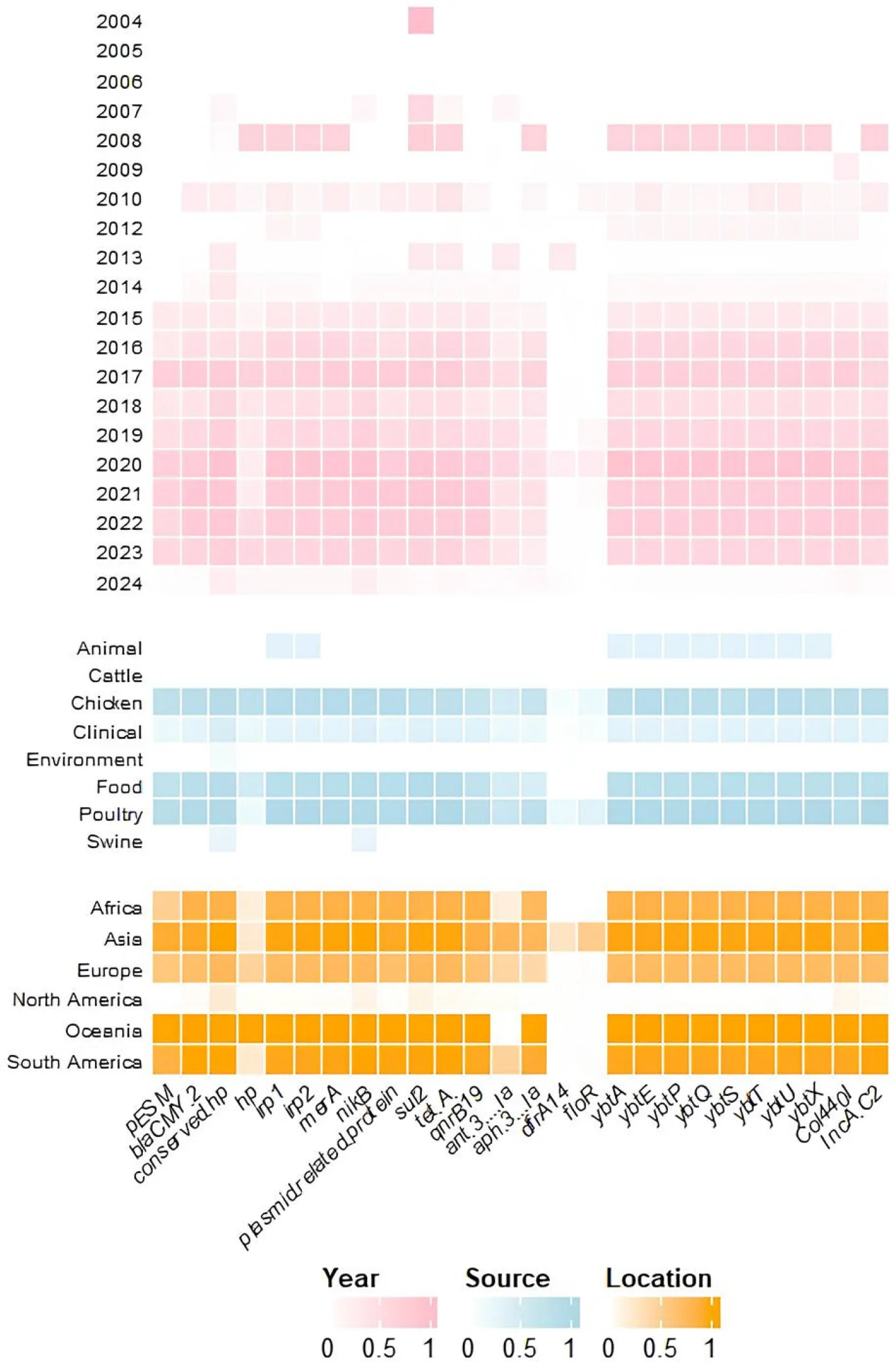
Year, source, and geographic distribution of target pESM genes. Heatmap on the distribution of specific targets across years (light pink), isolation sources (light blue), and geographic locations (orange). Color intensity represents the relative frequency of detection, as indicated by the gradient scale.

It is possible that these genes were allocated into pESM-positive genomes over the years. We evaluated the prevalence of target genes of pESM-positive genomes and other resistance and virulence genes while considering the year of isolation, source of isolation, and location (Figure 3). The pESM-positive genomes emerged from 2015 onwards; however, both the *mer* operon and *ybt* operon were already present in *Salmonella* Minnesota between 2006 and 2010, probably being carried by a megaplasmid precursor or integrated into the chromosome. From 2015, *mer*, *ybt*, *sul2*, *tet*(A), and *bla*_CMY-2_ began to emerge significantly, together with pESM, peaking between 2016 and 2020, when the highest number of isolates was observed. It can be inferred that these genes were inserted into pESM and began to disseminate more easily from this megaplasmid [54,55].

Thus, the increase in multidrug-resistant *Salmonella* Minnesota may be directly related to pESM in Brazil and in the other countries. In 2024, very few *Salmonella* Minnesota genomes were deposited in the NCBI database, limiting our ability to infer whether the distribution of pESM is increasing or decreasing (Figure 3).

### 3.3. Phylogenetics

**Figure 4:**
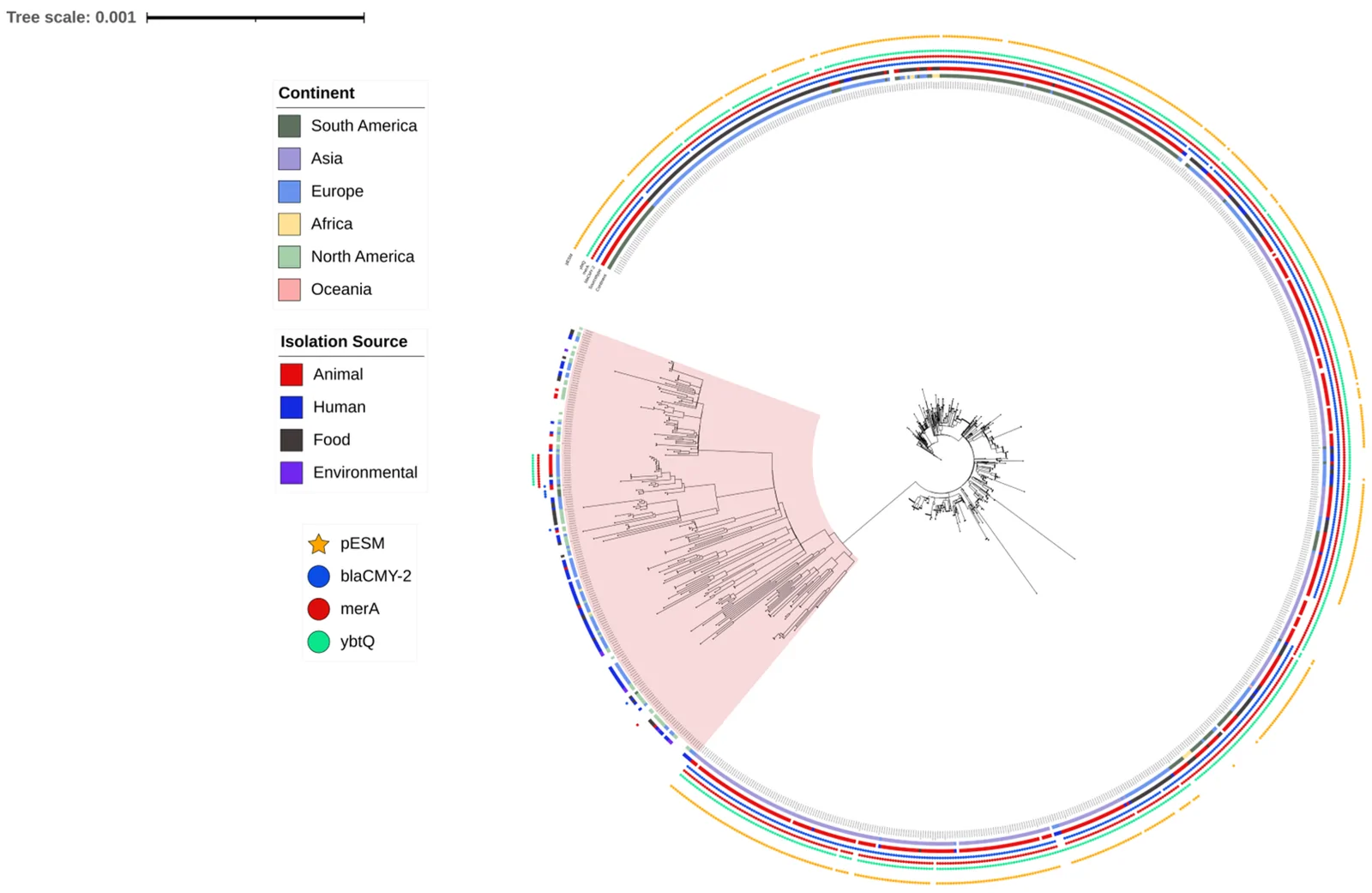
Maximum-likelihood SNP-based phylogeny of 935 *S.* Minnesota genomes using *S.* Minnesota ATCC 49284 as reference. The circular representation of the phylogeny was obtained using iTOL (http://itol.embl.de/ Accessed on 5 November 2024), ignoring branch lengths. The tree demonstrates the formation of a monophyletic group, highlighted in pink, clustering genomes lacking the putative pESM plasmid, which indicates that pESM-positive genomes, indicated by the yellow star, are phylogenetically closer to one another and likely belong to a specific lineage of *S.* Minnesota.

Concerning phylogenetics, we observed a clustering of pESM in genomes isolated from animals, mainly from Asia and South America, represented by Saudi Arabia and Brazil, respectively. A monophyletic group was formed among the genomes that did not present putative pESM and its main markers, including *bla*_CMY-2_ (Figure 4). Although most of the genomes belong to the same MLST, it can be noted that the monophyletic group represents an ancient lineage of emerging *S.* Minnesota since all were isolated before 2015 [56]. A previous study demonstrated through Bayesian analysis that the most likely common ancestor was before 1980 [8]. However, its emergence was only observed from 2010 onwards, being closely associated with the acquisition of persistence and antimicrobial resistance genes. As already demonstrated, most of the *S.* Minnesota genomes in the NCBI database come from Brazil and Saudi Arabia, mostly isolated from poultry, demonstrating the epidemiologic importance of this serovar in these countries. Phylogenetic analysis confirms that they are a part of the same lineage, presenting very similar characteristics, including putative pESM.

### 3.4. Salmonella Serovar, MLST, and Plasmid Replicons

A total of 1009 genomes were downloaded and assembled from the NCBI database. Of these, 935 passed the SISTR quality control and were confirmed as *S.* Minnesota. Ten MLSTs were identified among the 935 *S.* Minnesota: ST548 (*n* = 912), ST11 (*n* = 1), ST285 (*n* = 6), ST3088 (*n* = 4), ST2556 (*n* = 2), ST4923 (*n* = 2), and ST9594 (*n* = 1). Among those, only ST548 and ST9594 presented pESM. There is a certain dominance of sequence type 548 compared to the other few sequence types identified in this study. This specific ST represents the main lineages spreading in Brazil and worldwide [1,46], many of which carry pESM [8,57,58]. This dominance infers that pESM-positive genomes may be spreading through clonal expansion, not necessarily through horizontal gene transfer. ST9594 is not widespread, so there are no studies addressing it, and the corresponding genome was isolated from food in the United Kingdom in 2022. Therefore, there are not enough studies to infer that pESM is being transferred to other strains; however, this hypothesis cannot be ruled out. As observed with ST548, it can undergo clonal expansion and spread efficiently in the environment once established [29].

Forty different incompatibility groups were identified (Supplementary Table S2) [59]. Among these, the most frequently found were IncA/C2 (79.79%), followed by Col440I (74.97%), IncR (9.95%), IncI-1 (6.74%), ColpVC (8.02%), ColRNAI (5.45%), IncQ1 (4.71%), and IncFII(S) (4.28%). The predominance of the IncA/C2 incompatibility group is consistent with the high prevalence of putative pESM [60]. As previously stated, pESM is part of the IncA/C2 group, and the genomes carrying it usually also carry the PMQR *qnrB19* [8]. We identified the co-occurrence of IncA/C2 and *qnrB19* in 70.37% of the genomes analyzed in this study, and the chi-square test demonstrated significant association between *qnrB19* and pESM (Figure 2). Until 2019, *qnrB19* had not been reported in *S.* Minnesota from Brazil, and was first identified by Monte et al. (2019) [61]. *qnrB19* has become the most reported PMQR in Brazil and is also increasing in other countries, such as the United States [62]. Our results demonstrate the extent of quinolone resistance in the country, especially in strains that already carry resistance and virulence plasmids in poultry. The high risk that these pathogens represent highlights the need to strengthen surveillance measures in Brazil and develop actions to curb their dissemination. Certain incompatibility group markers, such as IncI1-1-Alpha, exhibited negative standardized residuals, suggesting mutual exclusivity with pESM.

## 4. Conclusions

Our findings provide evidence that the pESM megaplasmid plays an important role in modulating the recent epidemiology of *S.* Minnesota in poultry production systems, particularly in Brazil. The consistent detection of *bla*_CMY-2_, *sul2*, *tet(A)*, and the *mer* operon in pESM-positive genomes, as well as the yersiniabactin siderophore system, demonstrates that pESM is not only a multidrug resistance vector but also a reservoir of virulence and persistence determinants. These findings establish pESM as a critical vehicle for the global spread of antimicrobial resistance and virulence in *S.* Minnesota, particularly through food-producing animals. This scenario demands immediate action, including implementing targeted genomic surveillance programs, re-evaluating antimicrobial use in poultry, and taking proactive measures to limit environmental conditions selected for megaplasmid persistence and dissemination.

## Figures and Tables

**Table 1:** Target genes for identifying plasmid for emergent *S.* Minnesota (pESM) based on the Franco et al. [14] study.

Target	Protein/Function
*bla*_CMY-2_	β-lactamase; confers resistance to β-lactam antibiotics
Conserved-hp	Conserved hypothetical protein; unknown function but evolutionarily preserved
hp	Hypothetical protein; function not yet characterized
*irp1*	Siderophore yersiniabactin synthesis; involved in iron acquisition
*irp2*	Yersiniabactin biosynthesis/regulation; virulence factor
*merA*	Mercuric reductase; confers resistance to mercury compounds
*nikB*	Nickel transport system (NikABCDE); metal ion uptake
*trbA*	Regulator of plasmid transfer; involved in conjugation
*ybtP*	Yersiniabactin transport; part of the siderophore system
Plasmid-related protein	Plasmid-associated protein; possibly involved in mobility or replication

## Data Availability

The original data presented in the study are openly available in GenBank, and the accession numbers for each genome are provided in the Supplementary Material.

## Supplementary Materials

The following supporting information can be downloaded at https://www.mdpi.com/article/10.3390/genes17101230/s1, Table S1: Metadata of the *S.* Minnesota genomes analysed and downloaded from the Pathogen detection database from NCBI; Table S2: Frequency of the incompatibily groups identified for each *S.* Minnesota genome; Table S3: Genes frequencies n/N (%) within pESM-positive and pESM-negative isolates and hypothesis testing (Pearson Chi-Square test) restricted to genes absent from the pESM reference. p-BH: Benjamini–Hochberg adjusted *p*-value.
